# A systematic review on antimicrobial activities of green synthesised *Selaginella* silver nanoparticles

**DOI:** 10.1017/erm.2023.21

**Published:** 2023-08-03

**Authors:** Khushbu Wadhwa, Hardeep Kaur, Neha Kapoor, Soma M. Ghorai, Renu Gupta, Arunima Sahgal

**Affiliations:** 1 Ramjas College, University of Delhi, Delhi, India; 2 Hindu College, University of Delhi, Delhi, India

**Keywords:** Nanoparticles, pharmaceutics, secondary metabolites, *Selaginella*, therapeutics

## Abstract

**Background:**

Metallic nanoparticles from different natural sources exhibit superior therapeutic options as compared to the conventional methods. *Selaginella* species have attracted special attention of researchers worldwide due to the presence of bioactive molecules such as flavonoids, biflavonoids, triterpenes, steroids, saponins, tannins and other secondary metabolites that exhibit antimicrobial, antiplasmodial, anticancer and anti-inflammatory activities. Environment friendly green synthesised silver nanoparticles from *Selaginella* species provide viable, safe and efficient treatment against different fungal pathogens.

**Objective:**

This systematic review aims to summarise the literature pertaining to superior antifungal ability of green synthesised silver nanoparticles using plant extracts of *Selaginella* spp. in comparison to both aqueous and ethanolic raw plant extracts by electronically collecting articles from databases.

**Methods:**

The recommendations of the Preferred Reporting Items for Systematic Reviews and Meta-Analysis were taken into consideration while preparing this review. The titles and abstracts of the collected data were stored in Endnote20 based on the inclusion and exclusion criteria. The search strategy included literature from established sources like PubMed, Google Scholar and Retrieval System Online using subject descriptors.

**Results:**

The search yielded 60 articles with unique hits. After removal of duplications, 46 articles were identified, 40 were assessed and only seven articles were chosen and included in this review based on our eligibility criteria.

**Conclusion:**

The physicochemical and preliminary phytochemical investigations of *Selaginella* suggest higher drug potency of nanoparticles synthesised from plant extract against different diseases as compared to aqueous and ethanolic plant extracts. The study holds great promise as the synthesis of nanoparticles involves low energy consumption, minimal technology and least toxic effects.

## Introduction

Traditional herbal medicines exhibit great importance and are now being increasingly re-evaluated all over the world to determine their efficacy for the safer development of drug against different diseases. The heterosporous fern genus *Selaginella* is very rich in species (over 700), and is mainly found in the warm and moist climate having a worldwide distribution (Ref. 1). The desiccation-tolerant *Selaginella bryopteris* (L.) Bak. is an Indian resurrection and medicinal plant (Ref. 2). It belongs to the family Selaginellaceae and is commonly called ‘Sanjeevani’ for its therapeutic properties (Ref. 3). *Selaginella bryopteris* is found in the hilly terrain of Bihar, Jharkhand and Uttar-Pradesh. During monsoon season, it acquires a green appearance, while in dry season it undergoes extreme desiccation and revives only with return of favourable conditions, thus exhibiting resurrection capabilities. Most species of *Selaginella* however tolerate severe drought conditions due to the presence of trehalose; a disaccharide that provides structural and functional stability. In Indian folklore, *S. bryopteris* is reported to be a traditional herbal drug and considered as a tonic for revitalisation. The anti-cancer effect of *S. bryopteris* was determined in vitro against human hepatocellular carcinoma HepG2 cell line (Ref. 4). Many other species of *Selaginella* are well documented as Chinese traditional medicines such as *Selaginella doederleinii* Hieron. and *Selaginella pulvinata* (Hook. & Grev.) Maxim and are used to treat nasopharyngeal carcinoma and oesophageal cancer. Another herbal medicine found in China, *Selaginella tamariscina* (Beauv.) Spring, contains selaginellin derivatives that are rarely found in nature. However, clinical applications of *S. pulvinata*, were found to be very similar to those of *S. tamariscina*. Selaginellins are a small group of pigments that are exclusively found in the genus *Selaginella*. Since the first report of selaginellin from *Selaginella sinensis* (Desv.) Springs in 2007, more than 110 selaginellins, with different types of polyphenolic compounds, have been reported. Further, the secondary metabolites of resurrection plant species have also received special attention in the field of biotechnology and medicine. The major natural phytocompounds in the genus *Selaginella* are characteristic flavonoid-dimers (biflavonoids). The biflavonoids isolated from *S. tamariscina* show anti-inflammatory activity on lipopolysaccharide (LPS)-induced murine macrophages and colon epithelial cells (Ref. 5). Besides this, the use of *S. tamariscina* ethanol extract (STE) against glutamate-induced oxidative stress and neuronal cell death by controlling autophagy in a mouse hippocampal neuron cell line has also been reported (Ref. 6). One of the earliest reported articles on pharmacognostical studies of Indian *Selaginella* spp. was done by our group where we showed antimicrobial properties of its extracts (Ref. 7). Thereafter, our team worked on the synthesis of *Selaginella* silver nanoparticles by green method and observed much better antimicrobial properties in comparison to its raw extract (Ref. 8). Biosynthesised nanoparticles hold special mention in the category of phytomedicine due to their ease of production, lower cost, least toxicity and ecofriendly nature. To understand the superior antimicrobial potency of nano-formulations of *Selaginella,* identification of the early diagnostic markers and therapeutic targets is the primary step. This systematic review was hence designed to make a clear distinction between the competences of raw extract of *Selaginella viz a viz* the nano-drug prepared from its extract. We also propose that *Selaginella* nanoparticles might have a wide range of applications not only as anti-microbial drugs but also in the conservation of environment. Our claim is further substantiated by certain recent studies wherein green synthesised nanoparticles from *Selaginella* have shown excellent antimicrobial, anticoagulant and antiplatelet activities (Ref. 9).

## Materials and methods

This systematic literature review was conducted according to the recommendations of the Preferred Reporting Items for Systematic Reviews and Meta-Analysis guidelines.

### Search strategy

A search was performed using Google Scholar, Medline, Scopus and PubMed databases. The combination of the following keywords were selected and used for the search: *Selaginella* derived nanoparticles, bio efficacy of silver nanoparticles and phytochemistry of genus *Selaginella*. Articles that might be missed during various database searches were identified from the reference list of articles that were retrieved from initial search and were added to the list of selected articles.

### Eligibility criteria

Articles, titles and abstracts of literature paper were manually screened to exclude the data not related to the study. Relevant articles were studied to determine the eligibility criteria of this review. The following inclusion criteria were applied to select the studies: (1) only articles written in English; (2) observational original studies; (3) studies on *Selaginella* derived nanoparticles; (4) studies representing the bio-efficacy of nanoparticles derived from *Selaginella;* (5) studies that presented the aqueous and alcoholic extract of *Selaginella* and their various activities. The exclusion criteria were based on (1) general reviews on resurrection plants; (2) clinical reports and case studies; (3) book chapters, news, conference proceedings and editorial letters.

## Experimental

### Study selection and data collection

The identification of eligible criteria was performed by reading the titles and abstracts. The screening was performed by all the authors independently. The eligible full articles were then read by the authors and the inclusion and exclusion criteria were applied again. The included articles were determined by consensus among the authors and were critically appraised to assess the quality of the studies. The following data were extracted from the included articles: authors, year of publication, study design, bio-efficacy of *Selaginella* species, chemical extracts of *Selaginella* and its pharmaceutics, chemistry of various bioactive compounds in the extracts, *Selaginella*-derived nanoparticles and their bio-efficacy as targeted drug delivery agents (Table 1).

**Table 1.** Reference articles selected for study and data collection

Articles related to	Reference number
Genus *Selaginella*	(Refs 1, 2, 3, 10, 11, 12, 13, 14, 15)
Medicinal properties of genus *Selaginella*	(Refs 5, 16, 17, 18, 19, 20, 21, 22, 23, 24, 25, 26, 27, 28)
Aqueous and ethanolic extracts of *Selaginella* showing bio-efficacy	(Refs 7, 29, 30, 31)
*Selaginella* based nanoparticles	(Refs 8, 9, 32, 33, 34, 35, 36)
Commercially based nano-formulations/nanodrugs of *Selaginella*	(Refs 37, 38, 39)

### Data analysis

Microsoft Excel was used to tabulate the collected data. Various *Selaginella* species were classified according to their usage as traditional drugs in the Indo-pacific part of the world; mainly India, China, Sri Lanka and Indonesia (Table 2). In order to answer the objectives of this systematic review, a ‘directly proportional’ relationship was considered when the correlation analyses were positive, demonstrating the advantage of nano-synthesis of *Selaginella* over raw extracts. On the other hand, they were considered ‘inversely proportional’ when the correlation analyses were negative, that is, when very few reports (only three) were found for bio-efficacy of *S. bryopteris* nanoparticles, and the rest were for raw extracts that possess poor solubility and bioavailability capabilities. Eligible articles were analysed in detail using the content analysis method without any statistical tests.

**Table 2.** Summary of various *Selaginella* spp. and their important natural bioactive compounds found in different parts of the world

Provinces	*Selaginella* spp. studied	Year of study	Compounds derived from *Selaginella* spp.	Studies on crude extract of *Selaginella* spp.	*Selaginella* derived nanodrugs	Reference number
Korea	*S. tamariscina*	2018	Biflavonoids and its anti-inflammatory activities via ERK 1/2 signaling.	-----	-----	(Ref. 5)
Delhi, India	*S. bryopteris*	2020	-----	-----	Silver nanoparticle (AgNP) synthesised from *S. bryopteris* plant extract.	(Ref. 8)
Karnataka, India	*S. bryopteris*	2019	-----	-----	Silver nanoparticle (AgNP) synthesised from *S. bryopteris* plant extract.	(Ref. 9)
China	*S. pulvinata*	2010	Selaginellin derivatives exhibited antimicrobial activity against *C. albicans* and *S. aureus*.	Six selaginellin derivatives were isolated from ethyl acetate (EtOAc) extract, including three new analogues.	-----	(Ref. 10)
South Korea	*S. tamariscina*	2008	Isocryptomerin, exhibited antifungal activity.	-----	-----	(Ref. 16)
Saudi Arabia	*S. repanda*	2021	Phenols like coumarin, chlorogenic acid, caffeic acid, 4-methoxycinnamic acid. Flavonoids like rutin, vitexin, quercetin, kaemferol, apigenin, alkaloids, vitamins, terpenoids are identified.	Bioactive potential (antibacterial, antioxidant and anticancer) activities of ethanolic crude extract of *S. repanda* were studied.	-----	(Ref. 17)
Assam, Mizoram, India	*S. bryopteris*	2018	-----	-----	Silver nanoparticle (AgNP) synthesised from *S. bryopteris* plant extract	(Ref. 32)
Varanasi, India	*S. bryopteris*	2015	*β*-sitosterol, heveaflavone, syringaresinol, amentoflavone, *β*-sitosterol-*β*-D-glucoside, vanillic acid.	Hexane and acetone extract of *S. bryopteris* plant extract.	-----	(Ref. 18)
China	*S. convolute*	2019	-----	-----	Zinc oxide (ZnO) NP derived from leaf extract of *S. convolute*	(Ref. 33)
China	*S. tamariscina*	2020	-----	-----	Haemostatic NP derived from *S. tamariscina* carbonista (STC)	(Ref. 34)
West Java, Indonesia	*S. doederleinii*	2016	-----	-----	NP derived from leaf extract of *S. doederleinii*.	(Ref. 35)
South Africa and Douala	*S. myosurus*	2018	-----	-----	Synthesis of AgNP using aqueous extract of *S. myosurus* exhibited anti-inflammatory activity.	(Ref. 36)
China	*S. doederleinii*	2019	New oral drug delivery formulation of total biflavonoid extract (amentoflavone, robustaflavone, delicaflavone, 2″,3″-dihydro-3′,3″′-biapigenin, 3′,3″′-binaringenin) from *S. doederleinii*	-----	-----	(Ref. 39)
China	*S. sinensis*	2019	Sinesiols B-G, six novel neoligans are screened for their cytotoxicity activity against A549 and HepG2 human cancer cell lines.	Three new sesquilignans dimer of dihydrobenzo (b) furan moiety obtained from ethanolic extract studied for its cytotoxic activity.	-----	(Ref. 13)
China	*S. tamariscina*	2010	-----	Amentoflavone and extract of *S. tamariscina* screened for anti-tumor activity against cancer cell lines.	-----	(Ref. 19)
Korea	*S. tamariscina*	2007	Amentoflavone derived from *S. tamariscina* exhibits anti-candidal effect.	Methanolic extract of *S. tamariscina* were prepared.	-----	(Ref. 20)
Korea	*S. tamariscina*	2006	Amentoflavone exhibited antifungal activity against *C. albicans* by inducing the accumulation of trehalose in *C. albicans*.	Ethyl acetate extract of *S. tamariscina* yielded amentoflavone	-----	(Ref. 21)
Korea	*S. tamariscina*	2020	Amentoflavone, active compound of *S. tamariscina* inhibit in vitro and in vivo metastasis of cancer cells.	Aqueous extract of *S. tamariscina* prepared in distilled water to study its ability to inhibit migration and invasion of A459 and HT29 cancer cell lines.	-----	(Ref. 22)
Graz, Austria	*S. bryopteris*	2008	Antiplasmodial and leishmanicidal activity of bioflavonoids of *S. bryopteris.*	From ethanolic extract of *S. bryopteris*, different fractions were prepared such as EtOAc, n-Bu-OH. EtOAc shows highest activity against *Trypanosoma cruzei* and *Leishmania donovani*.	-----	(Ref. 23)
China	*S. doederleinii*	2013	Antiproliferation effect of six biflavonoids (Amentoflavone, robustaflavone, 2″,3″-dihydro-3′,3″′-biapigenin, 3′,3″′binaringenin, heveaflavone, 4′,7″,4″′-tetra-O-methyl-amentoflavone	Dichloromethane and acetate ether extract shows good cytotoxic activity against cancer cell lines.	-----	(Ref. 24)
Brazil	*S. convoluta*	2018	Flavonoids represent the bioactivities.	*S. convolut*ahexane (Sc-Hex), chloroform (Sc-CHCl_3_) and ethyl acetate (Sc-Ac0Et) extract were used to study antioxidant, antibacterial and cytotoxic activity.	-----	(Ref. 25)
Lucknow, U.P, India	*S. bryopteris*	2017	Extract were used to study anti-inflammatory phytomedicine for skin disease.	Aqueous, polar and non-polar methanolic extracts of *S. bryopteris.* Methanolic extracts were used for the treatment of 12-O-tetradecanoylphorbol-13-acetate (TPA) induced inflammation.	-----	(Ref. 26)
Taiwan	*S. tamariscina*	2007	Amentoflavone and apigenin were found to be the two compounds that reduces histamine release from mast cells and also reduced the release of IgE mediated hexosaminidase release as well as TNF-*α*, IL-4 production.	*S. tamariscina* extract (STE) prepared from ethanol exhibit antimetastatic activity on lung cancer cell line in vivo and in vitro.	-----	(Ref. 28)
China	*S. doederleinii*	2020	Twenty different flavonoids components were identified in the extract of *S. doederleini* ethyl acetate (SDEA) that inhibits growth of cancer cell lines such as A549, PC-9, CNE-2, MCF-7, HpG2.	Ethyl acetate of *S. doederleini* extract induces cell autophagic death and apoptosis in colorectal cancer cells.	-----	(Ref. 29)
Brazil	*S. sellowii*	2014	Amentoflavone and robustaflavone exhibited highest activity against *Leishmania amazonensis*	Antileishmanial activity was exhibited by the hydroethanolic extract derived from *S. sellowii.*	-----	(Ref. 31)

This particular research article mentions the strain name as *S. convolute* (Ref. 33).

## Results

**Figure 1.**
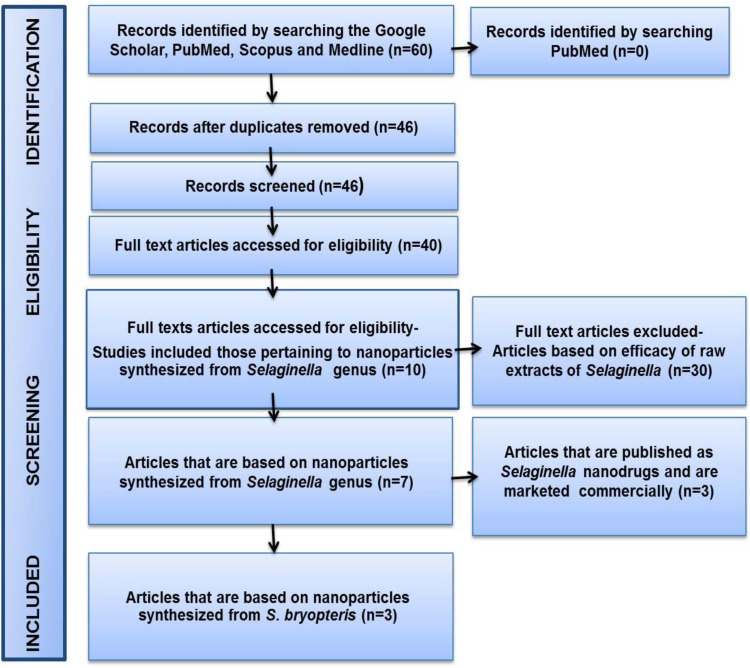
Flow diagram summarising systematic search and study selection.

The literature search identified a total of 46 articles in the Google Scholar, Medline, Scopus and PubMed databases that pertain to *Selaginella* spp. as medicinal plant, wherein *S. bryopteris* is an Indian medicinal plant. Among these 46 articles, 40 were eligible for reading the title and summary. Thirty articles showed differing bio-efficacy of raw extract of *Selaginella* but did not show results for our designed study. Therefore, a total of seven articles were included in this systematic review (Fig. 1). These seven articles were published between 2016 and 2020. All articles were observational cross-sectional studies and the methodological quality analyses indicated that 57.14% (*n* = 4) of the studies were considered with low risk of bias; and 42.86% (*n* = 3) were considered with medium/moderate risk of bias (Table 3). These seven articles also show different types of nanoparticles derived from various species of *Selaginella* such as *S. doederleinii* Hieron., *Selaginella myosurus* (SW.) Alston, *Selaginella convoluta* (Arn.) Spring and *S. bryopteris* (L.) Bak. (Refs 8, 9, 32, 33, 34, 35, 36). Nanoparticles derived from these species of *Selaginella* plant exhibited anti-cancer, anti-inflammatory, antimicrobial, anticoagulant, antiplatelet activities and also help in the management of pain. Only three articles clearly depicted the bio-efficacies shown by nanoparticles derived from *S. bryopteris*. Out of ten, three articles described the commercially marketed nanodrugs of *Selaginella*.

**Table 3.** Characteristics of the seven articles included in this study

Author name and reference number	Year	Nanoparticle synthesised from	Type of nanoparticles synthesised	Bio-efficacy of nanoparticles	Quality of studies
Yadav *et al*. (Ref. 8)	2020	*S. bryopteris*	Silver nanoparticle (AgNP)	Antimicrobial activity	Moderate risk of bias
Dakshayani *et al*. (Ref. 9)	2019	*S. bryopteris*	Silver nanoparticle (AgNP)	Antimicrobial, anticoagulant and antiplatelet activity	Moderate risk of bias
Biswas *et al*. (Ref. 32)	2018	*S. bryopteris*	Silver nanoparticle (AgNP)	Antimicrobial and photocatalytic activity	Moderate risk of bias
Xu *et al*. (Ref. 33)	2020	*S. convolute*	Zinc oxide nanoparticle (ZnO NP)	Pain management in emerging nursing care	Low risk of bias
Zhao *et al*. (Ref. 34)	2020	*S. tamariscina*	Haemostatic nanoparticles	For the treatment of haemorrhagic disease	Low risk of bias
Juniarti *et al*. (Ref. 35)	2016	*S. doederleinii*	Chitosan derived nanoparticles	Anti-cancer activity	Low risk of bias
Kedi *et al*. (Ref. 36)	2018	*S. myosurus*	Silver nanoparticle (AgNP)	Anti-inflammatory activity	Low risk of bias

## Discussion

This Study performed an analysis of the correlation between the bio-efficiency of nano-formulation derived from natural sources over the raw extracts from microbes. It lists and documents all the studies that have been done to characterise *Selaginella* derived natural phytochemicals with specific activities both as raw extracts as well as nano-formulations (Ref. 40). This study therefore provides an insight regarding future drug development where natural products can be explored as alternate medicines in many diseased conditions.

### Antimicrobial action of *Selaginella* extract

The compounds isolated from *Selaginella* belong to a wide class of alkaloids, benzenoids, chromones, coumarins, flavonoids, lignans, oxygen heterocycle, phenylpropanoids, pigments, quinoids, steroids, secolignans, neolignans and caffeoyl derivatives. The research on *S. pulvinata* extract revealed the presence of selaginellin derivatives, namely selaginellin G and selaginellin H, together with the biflavonoids and flavonoids. The presence of unique acetylene bond and *p*-quinone-methide functionalities in the selaginellin derivatives represents a rare group of naturally occurring phenolic compounds (Ref. 10). The Chinese medicinal plant, *S. tamariscina* contains a novel biflavonoid named isocryptomerin that exhibits antifungal and antibacterial activities on human pathogens, without any haemolytic effect on human erythrocytes (Ref. 16). Few others like *S. tamariscina* and *S. pulvinata* are very useful to promote blood circulation, while *S. doederleinii* is used as an antibacterial agent and to cure cardiovascular diseases. The antifungal effect of amentoflavone, another compound extracted from *S. tamariscina,* has paved the way for scientists to explore the antimicrobial properties in *S. bryopteris*, against bacterial and fungal pathogens such as *Staphylococcus aureus* and *Candida albicans.*

### Antioxidant, anti-inflammatory, anticancer and cardio and neuro-protective role of *Selaginella* extract

**Figure 2.**
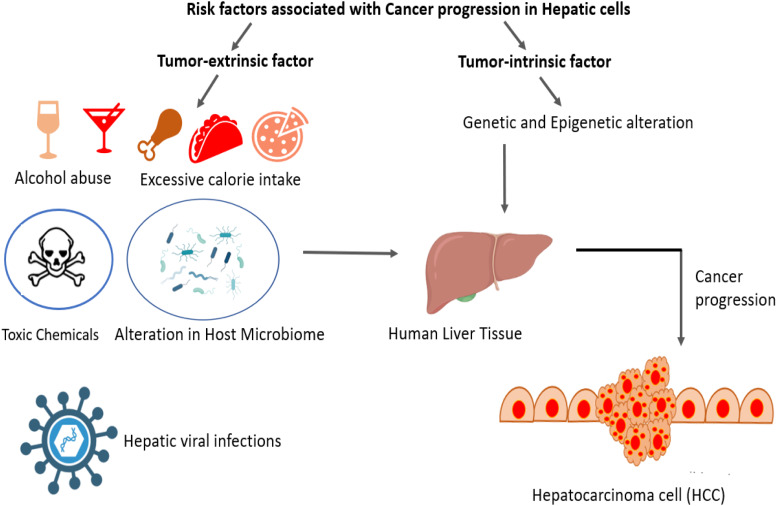
Schematic representation of the progression of cancer in human liver tissue.

In recent years, medicinal plants have received more attention owing to their pharmacological profile and easy availability to common people. Hepatocellular carcinoma (HCC) is considered as most common and leading cause of cancer-related mortality worldwide. The anti-cancer effect of *S. bryopteris* was evaluated by Pal *et al*. (Ref. 4), using a human hepatocellular carcinoma HepG2 cell line. Gas chromatography-mass spectrometry (GC-MS) was performed to analyse the phytochemicals present in *S. bryopteris* extract (SBE). The major bioactive phytocompounds identified by GC-MS were imidazole, amentoflavone, gallic acid, catechine, palmitic acid, lupeol, L-fucitol and myo-inositol. The anti-proliferative activity of SBE was evaluated by using MTT assay and further determined by change in the mitochondrial membrane potential, reactive oxygen species (ROS generation) and alteration in nuclear morphology (Fig. 2). A study by Jeong *et al*. (Ref. 6) have demonstrated the role of STE in inhibiting autophagy that may be useful for preventing and treating neurodegenerative diseases. Oxidative stress due to excessive production of ROS is considered as major cause of neurodegenerative disorders, including Parkinson's disease, Alzheimer's disease and cerebral ischemia. Prolonged oxidative stress leads to autophagy process that further results in autophagic cell death in the neuronal system. Administration of STE extract decreases the glutamate-induced cytotoxicity in HT22 cells via inhibition of ROS production and apoptosis (Ref. 6).

**Figure 3.**
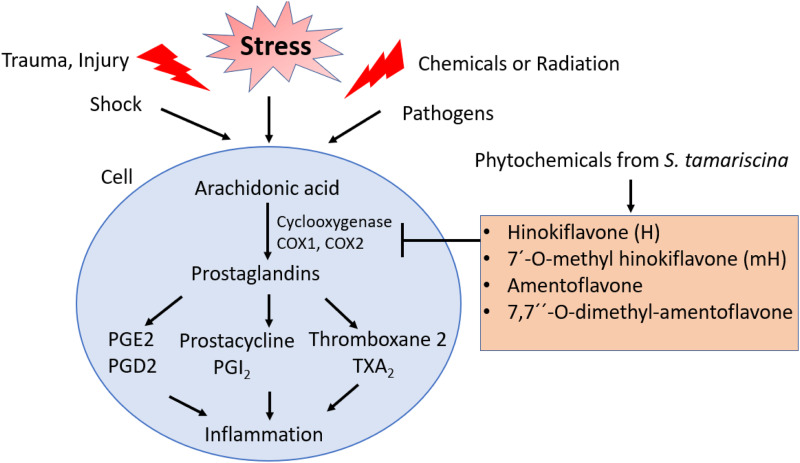
Schematic representation of the anti-inflammatory effect of *S. tamariscina* phytochemicals against cyclooxygenase enzymes; Cyclooxygenase 1 (COX1), Cyclooxygenase 2 (COX2).

*S. tamariscina* has been used for the treatment of dysmenorrhoea, amenorrhoea, haematuria, chronic hepatitis and hyperglycaemia. The study by Shim *et al*. (Ref. 5) demonstrated the anti-inflammatory activity of two biflavonoid compound called as Hinokiflavone (H) and 7′-O-methylhinokiflavone (mH). They also examined their activity in LPS-mediated murine macrophages (RAW 264.7) and colon epithelial cells (HT-29). Both H and mH can suppress the production of LPS-induced expression of inducible nitric oxide synthase (iNOS) and cyclooxygenase (COX-2) and can lead to activation of nuclear factor-*κ*B (NF-*κ*B) and extracellular regulated kinases (ERK), thus inhibiting inflammation (Fig. 3).

A study by Adnan, *et al*. (Ref. 17) has demonstrated the phytochemistry of *S. repanda* (Desv. & Poir.) Spring. ethanolic crude extract using Ultra High Performance Liquid Chromatography–PDA detector mass spectrophotometer. The study has revealed the presence of various phenols such as chlorogenic acid, 7-hydroxycoumarine, 2-amino-1,3,4-octadecanetriol, 4-coumaric acid, 4-methoxy cinnamic acid, caffeic acid, coumarin, isoferulic acid and formononetin. The flavonoids compounds identified in this study are rutin, apigenin, vitexin, genistein, glycitin, luteolin, rhamnetin, kaempferol, diosmetin, quercetin, quercetin-3*β*-D-glucoside and glycitein. The identified phytochemicals exhibit therapeutic applications including antioxidant, anti-inflammatory, anticancer and cardio-protective effects.

A study by Muema *et al*. (Ref. 41) has evaluated the anti-proliferative activities of both Dichloromethane (DCM) and ethyl-acetate (EA) extract of *S. doederleinii* on three cancer cell lines HT-29, He-La and A549 at different concentration ranging from 12.5 to 200 *μ*g/ml. The EA fraction exhibited anti-proliferative activity against the HT-29, He-La cell lines by inhibiting the cell growth rate in a dose-dependent manner with IC_50_ values of 55.6 ± 1.3 and 69.2 ± 1.3 *μ*g/ml, while the DCM fraction exhibited the activity against A549 cell line with an IC_50_ value of 55.9 ± 12.6 *μ*g/ml. Furthermore, phytochemical investigation of DCM extracts has led to the identification of 7″-methyl ether tetrahydrohinokiflavone that exhibited the strongest activity against all three cell lines by inhibiting the cell growth rate in a dose-dependent manner.

### Role of green synthesised nanoparticles from *Selaginella*

Among the articles in literature, many provide strong evidence of therapeutic and medicinal value of *Selaginella* and show a directly proportional interaction between raw extracts and their bio-efficacy with respect to antimicrobial and anti-inflammatory properties. On the other hand, very few articles showed that silver nanoparticles synthesised from the plant extract of *Selaginella* exhibits enormous therapeutic applications. The plant extract of *Selaginella* acts as a capping and reducing agent (Ref. 9). Besides containing various secondary metabolites such as carbohydrates, chromones, flavonoids, steroids, quinoids, lignans, the raw extract of *Selaginella* also contains functional organic groups like -OH, -COOH, C-C, C-O containing coumarins (Ref. 11). These secondary metabolites exhibit ligation properties by binding to metal ions and get reduced to form metal nanoparticles. For the green synthesis and determination of cytotoxicity of silver nanoparticles, several factors must be considered including chemical composition, surface chemistry, size and its distribution, coating/capping, agglomeration, dissolution rate, particle morphology and reactivity in solution, efficiency of ion release and the type of reducing agents (Ref. 42).

The small number of screened articles evaluated the antimicrobial activity of *Selaginella*-derived silver nanoparticles by using agar disc-diffusion methods against bacterial and fungal pathogens. To study the antimicrobial activity of SPE-Ag-NPs (*Selaginella* plant extract Ag-NPs), Ciprofloxacin and Amphotericin B were used as standard drugs and these nanoparticles were examined by measuring the diameter of the zone of inhibition around each well (Ref. 32). The zone of inhibition and MIC values were determined for plant extract as well as for crude compounds against the gram-positive and gram-negative bacteria and also against *Candida* species (Ref. 18). The radial growth inhibition zone exhibited an increase with enhanced concentration of SPE-AgNPs, thus evincing their potent antimicrobial activity as compared to the raw extracts from *Selaginella* or the traditionally used drugs.

The articles included in this systematic review also analysed the functional capability of other nanoparticles, such as Zinc oxide derived nanoparticles (ZnO NPs) – which demonstrate considerable biological activities. The ZnO NPs prepared from leaf extracts of *S. convoluta* show good potency as anti-nociceptive and a muscle relaxant, thus holding promise in pain management as an emerging nursing care in the future (Ref. 33). Also, articles were included which demonstrated that nanoparticles synthesised by charcoal processing of *S. tamariscina,* called *S. tamariscina carbonisatus* (STC), act as a type of calcined herb-drug to promote haemostasis. The effects of STC-NPs showed low toxicity in the hemorheological and coagulation systems and can be a promising candidate for biological application. This haemostatic effect of STC-NPs suggests a new approach for the development of therapeutic drugs that can be used in the management of haemorrhagic diseases (Ref. 34). It has also been reported that nanoparticles synthesised from *S. doederleinii* possesses anti-cancer activity due to the presence of phytochemical compounds, especially flavonoids such as flavones (apigenin) and flavonones (naringenin) (Ref. 35). Additionally, silver nanoparticles synthesised from *S. myosurus* by green, novel and environmentally friendly pathway exhibit an anti-inflammatory activity by inhibiting the denaturation of albumin protein; which is the major cause of tissue damage in arthritic reactions during inflammation (Ref. 36).

### Drug delivery system for better action of bio-formulations

Few articles were worth considering for their reference of drug delivery systems especially through the oral route owing to the safety and simplicity of their administration. Nevertheless, several new formulations such as liposomes, nanoparticles, self-nano emulsifying drug delivery systems and solid dispersions are recommended to enhance the aqueous solubility and oral bioavailability of some compounds. Among these preparations, solid dispersion is one of the most fascinating formulations that improve drug release, enhance gastrointestinal absorption and oral bioavailability (Ref. 43). Amorphous solid dispersions (ASDs) are defined as molecular mixtures of hydrophobic drugs with a hydrophilic carrier that can provide more solubility over the crystalline form (Ref. 44). ASDs are generally prepared from polymers, specifically a hydrophobic polymer that is capable of inhibiting drug crystallisation and helping in better drug release. Hinokiflavone (HF), a biflavonoid isolated from the medicinal plant *S. tamariscina* (Ref. 12) has been shown to have antitumor capacity but possesses poor solubility and low bioavailability that restricts its use for medicinal purposes. To overcome these challenges, Polyvinyl caprolactam-polyvinyl acetate-polyethylene glycol (Soluplus®) is used which is an amphiphilic polymer that has the capacity to increase the solubility of hydrophobic drugs. This polymer helps in the formation of a micellar-like structure in a solution having a low critical micelle concentration (CMC). The molecular structure of Soluplus contains a hydrophilic portion of polyethylene glycol backbone and a hydrophobic portion of ethylene caprolactam/vinyl acetate side chain (Ref. 37). The use of D-*α*-Tocopherol acid polyethylene glycol 1000 succinate (TPGS), the derivative of vitamin E, a non-ionic surfactant containing the hydrophilic head and lipophilic tail helps to improve drug solubility and drug encapsulation efficiency. The physicochemical properties of HF hybrid micelles are investigated including particle size, zeta potential, encapsulation efficiency, drug loading, CMC value and stability. Nevertheless, HF hybrid micelles exhibit improved anticancer activity and induce mitochondrial-mediated apoptosis by increasing the levels of ROS. They may be considered as a potential drug delivery system for the treatment of lung adenocarcinoma (Ref. 38). Similarly, amentoflavone, robustaflavone, 2″,3″-dihydro-3′,3″′-biapigenin, 3′,3″′-binaringenein and delicaflavone are the five major components in the total biflavonoid extract from *S. doederleinii* (TBESD) that display anticancer properties. To ameliorate the solubility, dissolution rate and bioavailability of TBESD, a new oral drug delivery formulation has been made with the help of a solid dispersion technique. TBESD amorphous solid dispersion (TBESD-ASD) with polyvinylpyrrolidone K-30 was prepared by using the solvent evaporation technique which showed higher solubility and dissolution rates as compared to raw TBESD (Ref. 39) and hence can prove to be a good promising chemotherapeutic agent for cancer treatment.

The solid dispersion nanoparticles with better dissolution and oral bioavailability might therefore prove to be a revolutionary drug delivery system with much better technological advantage as compared to conventional delivery systems.

Limitation: As is true for any research, this systematic review is not without limitations. There is publication bias that must be understood as certain articles (grey literature) were not included. The systematic review clearly has a language bias wherein the articles published in English language were only included. However the systematic review presented here gives a very affirmative role to nanoparticles as potent antimicrobial agents. Hence, this review can serve as a guide towards improving antimicrobial strategy with minimal toxicity.

## Conclusion

The study shows that many species of the genus *Selaginella* are a rich source of various bioactive compounds like flavonoids and biflavonoids. Flavones such as apigenin, luteolin and diosmetin are ubiquitously found in many other popular plant species (Ref. 45), including *Selaginella* spp. Specific flavonoids such as amentoflavone are also found to be potent antifungal agent (Ref. 46). All the above-mentioned flavones found in *Selaginella* spp are known to possess anticancer, antimicrobial, anti-inflammatory and many other pharma nutraceutical properties. However, very few articles have emphasised the superior role of nano-formulations made from the raw extracts of *Selaginella*. Nevertheless, the review of such articles has been very promising highlighting a much better antimicrobial activity of nanoparticles over raw extract. Still more research is needed to further explore the mechanism of action of these compounds and their bioactivities. Nanoparticles synthesised from *Selaginella* hold promise not only in clinics and pharmaceutics but also in other fields like restoration of old heritage buildings and conservation of environment. Since, *Selaginella* AgNPs are an efficient anti-microbial and anti-fungal agent; they may be used to remove the fungal and algal growths over the monuments that are responsible for their rapid degradation. *Selaginella* silver nanoparticles can also be explored in cleaning water ecosystems, wetlands and other ecologically dynamic systems. The chemical and physical methods used for extractions of phytochemicals from *Selaginella* for its use as medicine involves production of many effluents that are ecologically harmful. Management of such processes for the potential risks needs deeper understanding to improve both nature and human lives. With better knowledge of the alternative synthesis methods, it would be possible to have the same drug quality of *Selaginella*, with minimal environmental hazards. Therefore, understanding the procedure that involves greener ways of drug synthesis with improved drug delivery system in the form of nano-formulations, as highlighted in this review, is critical. These findings may be utilised to improve the synthesis of nano-drugs from different *Selaginella* species which entails low cost, minimal technology, less harmful effluents and better drug efficacy.
